# Imaging Demyelinated Axons After Spinal Cord Injuries with PET Tracer [^18^F]3F4AP

**DOI:** 10.2967/jnumed.124.268242

**Published:** 2025-02

**Authors:** Karla M. Ramos-Torres, Sara Conti, Yu-Peng Zhou, Amal Tiss, Celine Caravagna, Kazue Takahashi, Miao He, Moses Q. Wilks, Sophie Eckl, Yang Sun, Jason Biundo, Kuang Gong, Zhigang He, Clas Linnman, Pedro Brugarolas

**Affiliations:** 1 Department of Radiology, Massachusetts General Hospital and Harvard Medical School, Boston, Massachusetts;; 2 F.M. Kirby Neurobiology Center, Boston Children’s Hospital and Harvard Medical School, Boston, Massachusetts; and; 3 Department of Physical Medicine and Rehabilitation, Spaulding Rehabilitation Hospital and Harvard Medical School, Boston, Massachusetts

**Keywords:** spinal cord injury, demyelination, [^18^F]3F4AP, PET, translational study

## Abstract

Spinal cord injuries (SCIs) often lead to lifelong disability. Among the various types of injuries, incomplete and discomplete injuries, where some axons remain intact, offer potential for recovery. However, demyelination of these spared axons can worsen disability. Demyelination is a reversible phenomenon, and drugs such as 4-aminopyridine (4AP), which target K^+^ channels in demyelinated axons, show that conduction can be restored. Yet, accurately assessing and monitoring demyelination after SCI remains challenging because of the lack of suitable imaging methods. In this study, we introduce a novel approach using the PET tracer, 3-[^18^F]fluoro-4-aminopyridine ([^18^F]3F4AP), specifically targeting K^+^ channels in demyelinated axons for SCI imaging. **Methods:** Rats with incomplete contusion injuries were imaged with [^18^F]3F4AP PET up to 1 mo after injury, followed by further validation of PET imaging results with autoradiography and immunohistochemistry of postmortem spinal cord tissue. A proof-of-concept study in 2 human subjects with incomplete injuries of different severities and etiologies was also performed. **Results:** [^18^F]3F4AP PET of SCI rats revealed a more than 2-fold increase in tracer binding highly localized to the injured segment of the cord at 7 d after injury relative to baseline (SUV ratio = 2.49 ± 0.09 for 7 d after injury vs. 1.14 ± 0.10 for baseline), revealing [^18^F]3F4AP’s exceptional sensitivity to injury and its ability to detect temporal changes. Autoradiography, histology, and immunohistochemistry confirmed [^18^F]3F4AP’s targeting of demyelinated axons. In humans, [^18^F]3F4AP differentiated between a severe and a largely recovered incomplete injury, indicating axonal loss and demyelination, respectively. Moreover, alterations in tracer delivery were evident on dynamic PET images, suggestive of differences in spinal cord blood flow between the injuries. **Conclusion:** [^18^F]3F4AP demonstrates efficacy in detecting incomplete SCI in both animal models and humans. The potential for monitoring post-SCI demyelination changes and response to therapy underscores the utility of [^18^F]3F4AP in advancing our understanding and management of SCI.

## Visual Abstract

**Figure:**
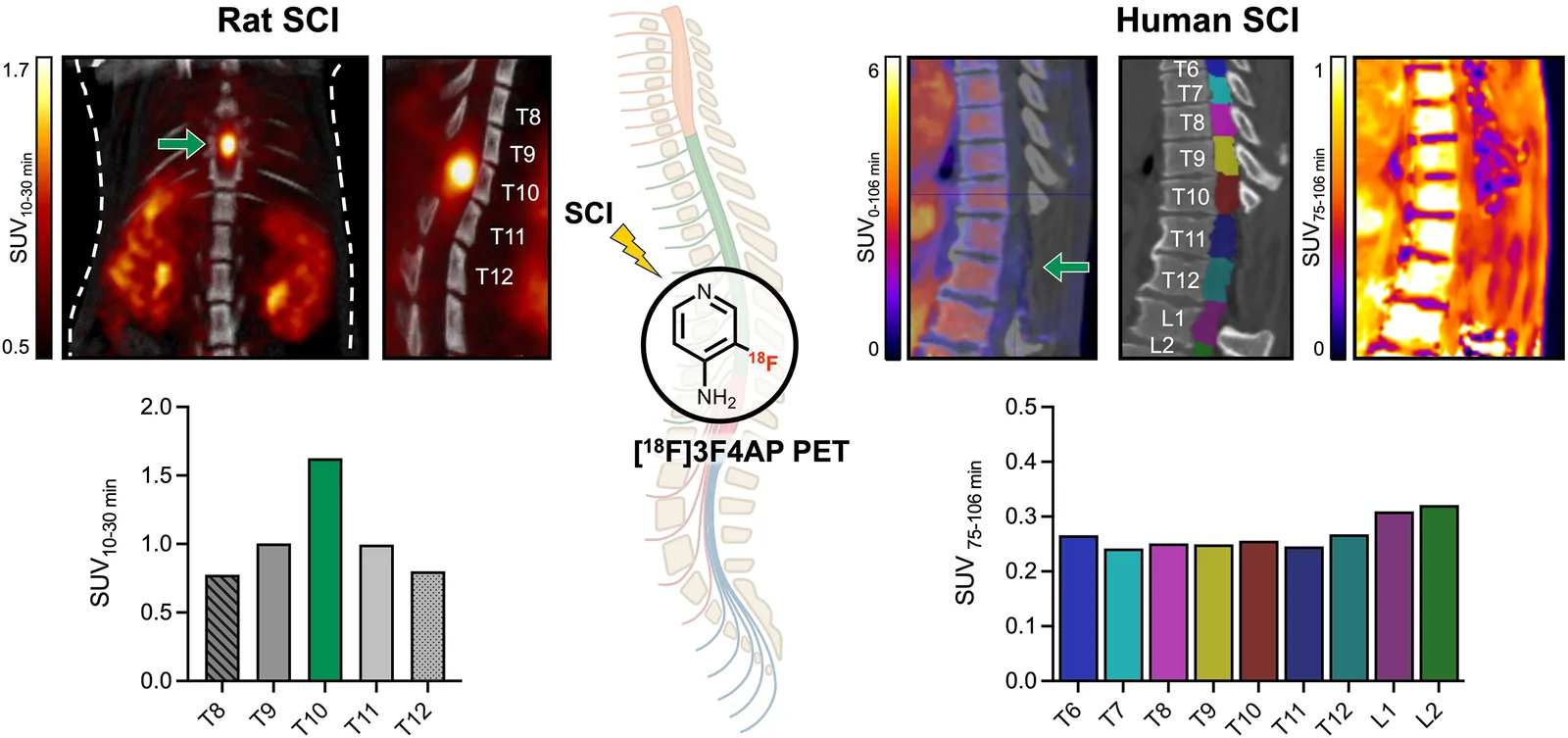

Spinal cord injury (SCI) can result in devastating loss of mobility, often causing lifelong struggles to regain independence and quality of life. During the initial months after injury, there are rapid degenerative changes, such as demyelination at and around the lesion, which are associated with a less favorable recovery outcome (1–3). Demyelination, which impairs axonal conduction, is believed to significantly contribute to disability after incomplete SCI (2,4). Furthermore, with the loss of myelin, spared demyelinated axons are more vulnerable to damage, potentially leading to further neuronal loss via necrosis or apoptosis. Consequently, therapeutic approaches that promote remyelination are a major research focus in SCI, with much knowledge gained from multiple sclerosis. These include pharmacologic, cell, physical, and electrical stimulation therapies (4–16). Although many of these approaches have shown promise in standardized animal models, a significant challenge in translating these treatments to humans is determining how to monitor their response, considering the heterogeneous nature of human SCI.

Existing MRI methods to track SCI progression rely on anatomic changes (17). These approaches lack specificity and exhibit modest correlation with clinical symptoms. In addition, the presence of stabilization hardware can create MR artifacts, restricting the feasibility of using MRI to directly assess characteristics near the spinal cord lesion (18). In contrast, PET has the potential to offer sensitive and quantitative imaging of biochemical changes after SCI. Various PET tracers have been used to evaluate the biologic and physiologic outcomes of SCI (19). These include [^18^F]FDG to assess metabolism (20), [^18^F]GE-180, a tracer that targets translocator protein 18, to examine neuroinflammation (21), [^11^C]-AFM, a presynaptic serotonin marker, to assess axonal connectivity across a lesion (22), and [^11^C]-UCB-J, a tracer for synaptic vesicle glycoprotein 2A, to assess synaptic density (23).

Since demyelinated axons may drive disability in SCI, imaging these fibers could potentially inform disease prognosis and treatment response. Myelin-binding radiotracers based on the diaminostilbene pharmacophore have been developed to evaluate myelin changes in the spinal cord in both experimental models of demyelination (24) and rodent SCI models (25,26). As an alternative approach, we have developed a novel radiotracer, 3-[^18^F]fluoro-4-aminopyridine ([^18^F]3F4AP), on the basis of the multiple sclerosis drug 4-aminopyridine (4AP, dalfampridine) that targets potassium (K^+^) channels in demyelinated axons (27). [^18^F]3F4AP has demonstrated high sensitivity to demyelinated lesions in animal models of multiple sclerosis (27) and is currently under investigation in people with multiple sclerosis (clinicaltrials.gov, NCT04699747). Additionally, it has shown high sensitivity to a minor traumatic brain injury in a rhesus macaque (28) as well as wide biodistribution and low radiation dosimetry in humans (29). Moreover, whereas 4AP has shown efficacy in animal models (30–34), studies evaluating it in people after SCI have yielded mixed results (35–43), raising the question of who may benefit from a therapy designed to enhance conduction of demyelinated fibers.

Given [^18^F]3F4AP’s sensitivity to traumatic injury, the potential therapeutic response of its parent compound, and the well-documented changes in axonal K^+^ channel expression in SCI models (44,45), we explored its use for imaging SCI. Specifically, this work examines the changes in [^18^F]3F4AP uptake in and around the injured cord in a well-established model of incomplete SCI over time, correlating these changes with histologic alterations in myelin and the functional in vivo phenotype. Finally, we present the first images of [^18^F]3F4AP in humans with SCI, offering valuable insights into the possible clinical application of this novel PET radiotracer for monitoring and understanding SCI.

## MATERIALS AND METHODS

All details for the study design and methodology are presented in the supplemental materials (available at http://jnm.snmjournals.org) (46–48).

### Animal Studies

All rodent procedures were approved by the Institutional Animal Care and Use Committee at the Massachusetts General Hospital. All animal studies were conducted in compliance with the Animal Research: Reporting In Vivo Experiments guidelines for reporting animal experiments.

### In Vivo Evaluation of SCI in Rats with [^18^F]3F4AP

Rats were subjected to a moderate severity spinal contusion injury at the mid thoracic level (T10) as previously described (49). At various postinjury time points (baseline, 2, 7, 14, and 28 days postinjury [dpi]), animals were imaged by PET/CT. Clinical evaluation of SCI rats was performed throughout the study using the Basso, Beattie, and Bresnahan (BBB) locomotor rating scale (50). [^18^F]3F4AP was produced in a GE HealthCare TRACERlab FX2N synthesizer according to previously reported methods (51) and administered to rats via the tail vein under isoflurane anesthesia. Multibed position PET was acquired in a PET/SPECT/CT scanner (Triumph LabPET; Trifoil), followed by CT acquisition in a 2-bed position for anatomic reference. Full details for the animal injury model, clinical evaluation, radiotracer synthesis, image acquisition, reconstruction, and analysis are presented in the supplemental materials.

### Ex Vivo Evaluation of Rat SCI

Immediately after PET/CT acquisition, rats were euthanized; the spinal cord between approximately T1 and L5 was isolated and sectioned longitudinally at 20-µm sections for autoradiography. Corresponding autoradiography slides were selected for histologic staining of myelin. A separate cohort of animals was used to produce 40-µm sections for immunofluorescence staining. Full details are available in the supplemental materials.

### Human Studies

Human imaging studies were performed in line with the principles of the Declaration of Helsinki. Approval was granted by the Institutional Review Board at the Massachusetts General Hospital (IRB no. 2020P003898). [^18^F]3F4AP was administered under an investigational new drug authorization from the U.S. Food and Drug Administration (investigational new drug no. 135,532; sponsored by Brugarolas).

### Evaluation of Human SCI with [^18^F]3F4AP

[^18^F]3F4AP was produced by the Massachusetts General Hospital PET Core current good manufacturing practices radiopharmacy using a Neptis ORA synthesizer as previously communicated (29,52). The synthesis method is based on the previous report by Basuli et al. (51). Two male volunteers with SCI were imaged on a GE HealthCare Discovery MI PET/CT scanner. Low-dose CT of the injury was acquired, followed by dynamic PET acquisition from 0 to 45 min and from 75 to 106 min. One of the subjects underwent additional MRI on a Siemens 3 T MMR scanner using the body coils to visualize the lesion. Full details for the image acquisition protocols, processing, and analysis are provided in the supplemental materials.

### Statistical Analysis

Statistical analysis of in vivo PET was performed using GraphPad Prism (version 10.2). Descriptive statistics including mean, SD, and SE were calculated for each group. Two-group *t* tests and multigroup *t* tests (e.g., ANOVA) with a significance level α of 0.05 were used to assess differences among groups. Grouped data are reported as mean ± SEM.

## RESULTS

### Thoracic Spinal Contusion in Rats as a Model for SCI

**FIGURE 1.**
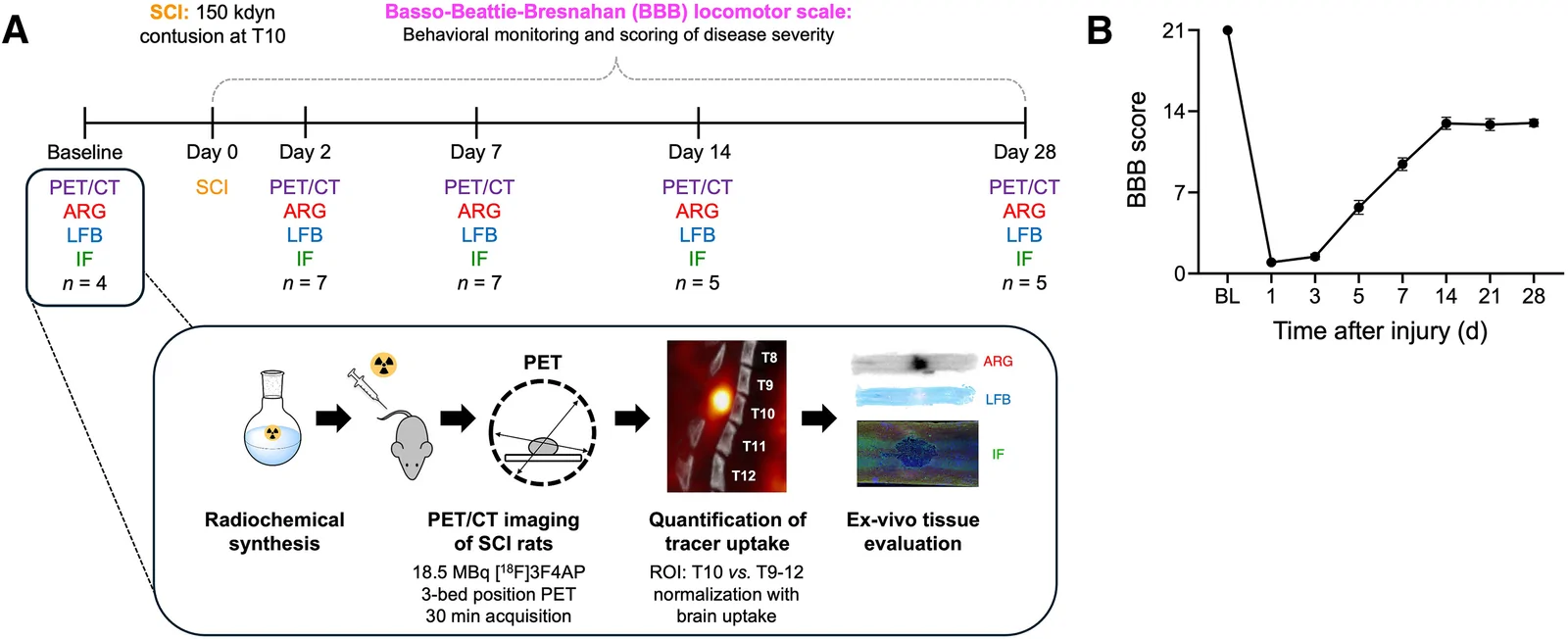
Cross-sectional evaluation of rat SCI with [^18^F]3F4AP. (A) Rats were subjected to spinal contusion injury and evaluated at different time points (baseline, 2, 7, 14, and 28 dpi) with [^18^F]3F4AP PET and postmortem evaluation of spinal cord tissue at each time point. (B) Clinical evaluation was performed at baseline and at 1, 3, 5, 7, 14, 21, and 28 dpi using BBB score. ARG = autoradiography; IF = immunofluorescence; ROI = region of interest.

Rodent models of SCI have been extensively used to examine various biologic processes (inflammation, demyelination, axonal loss, etc.) in different degrees of injury (53). In this study, we chose spinal contusion in rats as a model of incomplete injury to investigate whether PET imaging with [^18^F]3F4AP can detect spared demyelinated axons. Previous studies with this model have shown acute demyelination at the injury starting 7 dpi followed by slow remyelination (49). Additional studies in a related SCI compression model have shown large increases and redistribution of voltage-gated potassium channel (K_v_) K_v_1.1 and K_v_1.2 in the spinal cord white matter also starting at 7 dpi (45). For this purpose, adult female rats underwent force-controlled spinal contusion at T10 and were assessed at various time points after injury via [^18^F]3F4AP PET imaging. The spinal cord tissue was evaluated ex vivo by autoradiography, histologic staining for myelin, and immunohistochemical staining of myelin and axonal markers (Fig. 1A). Behavioral assessment using the BBB locomotor scale confirmed a sharp decrease in locomotion (BBB score) at 1 dpi that spontaneously improved until reaching a plateau 2 wk after injury (Fig. 1B). This result aligns with previously observed symptomatic demonstration in rats after a moderate-severity spinal contusion injury, where behavioral, anatomic (electron microscopy), and electrophysiologic (postinjury conduction) assessments showed a marked pattern of demyelination at 1 wk after injury (49). We therefore pondered if [^18^F]3F4AP could be used to detect spared demyelinated fibers around the peak of disease and after the initial inflammatory response has subsided in the subacute injury phase.

### Imaging Demyelinated Axons in Rats 7 Days After Spinal Contusion

**FIGURE 2.**
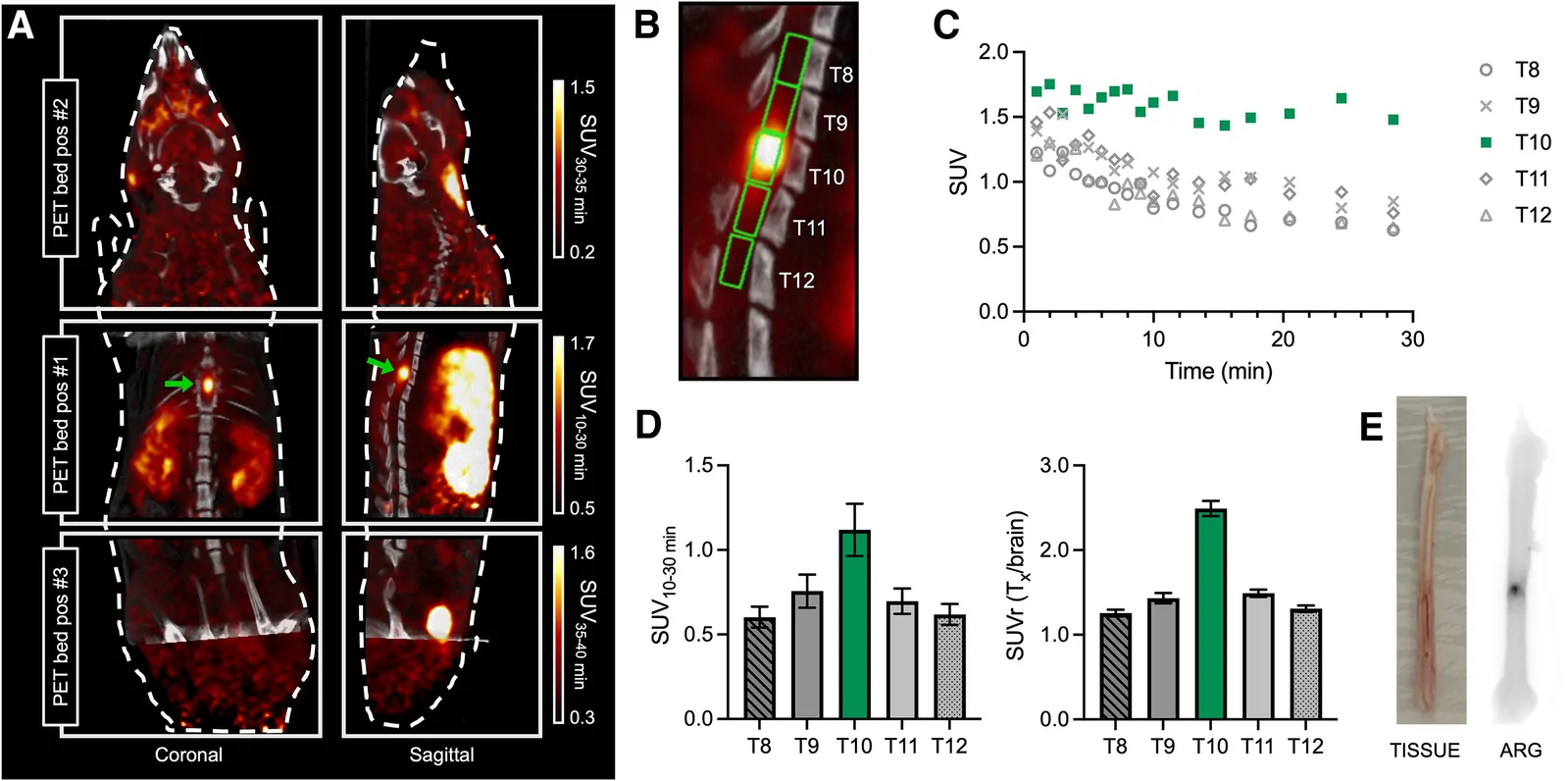
Evaluation of [^18^F]3F4AP in rodent SCI. (A) Representative coronal and sagittal views of whole-body (3-bed position) [^18^F]3F4AP PET/CT in rat at 7 dpi. (B) Magnification of PET/CT images showing laminectomy at T10, high PET signal in injured cord, and regions of interest selected for quantification. (C) Representative time–activity curves extracted from regions of interest corresponding to lesion site (T10, green) and surrounding vertebral segments (T8–T12). (D) Quantification of 10–30-min SUV and normalized vertebral SUV to brain uptake (*n* = 7). Data are mean ± SEM. (E) Explanted spinal cord (left) and corresponding autoradiography (ARG; right) of 1 rat. Representative PET images and ex vivo tissue correspond to different animals.

On the basis of previous reports of peak demyelination and conduction loss at 7 dpi (49), we first evaluated [^18^F]3F4AP binding at this time point. Rats were scanned in the supine position to minimize motion of the spine due to breathing during the scan using a multibed position acquisition protocol consisting of a 0–30-min dynamic scan of the trunk region, a 5-min static acquisition of the head, a 5-min static acquisition of the pelvic area, followed by a CT scan for anatomic reference. PET/CT images showed an area of high focal uptake at the site of injury, and magnification of the coregistered images confirmed that this uptake corresponded to the impact location beneath the laminectomy at T10 (Figs. 2A and 2B). Time–activity curves revealed slower tracer washout at T10, indicating greater tracer binding compared with adjacent segments (T8–T12) (Fig. 2C). On the basis of the time–activity curves, we selected the SUV from 10–30 min as a measure of binding and quantified the signal at the different spinal cord segments. The 10–30-min mean SUV at T10 was 1.12 ± 0.15 (*n* = 7), a 187% increase compared with that at T8 (0.60 ± 0.06). For further comparisons across animals, values were normalized to 30–35-min whole-brain SUV as an internal control, as previous dynamic studies in rats and mice have shown that the brain time–activity curve is stable during this period (27,54). Relative to the brain, T8 showed an SUV ratio (SUVr) of 1.26 ± 0.04 and the lesion showed a SUVr of 2.49 ± 0.09 (*n* = 7) (Fig. 2D). Notably, the high uptake at the injury could also be clearly observed in a standard clinical PET/CT scanner (Supplemental Fig. 1). Autoradiography of the extruded cord postmortem confirmed an area of very high focal uptake within the impacted cord (Fig. 2E).

### Imaging Injury Progression at Different Time Points After Spinal Contusion

**FIGURE 3.**
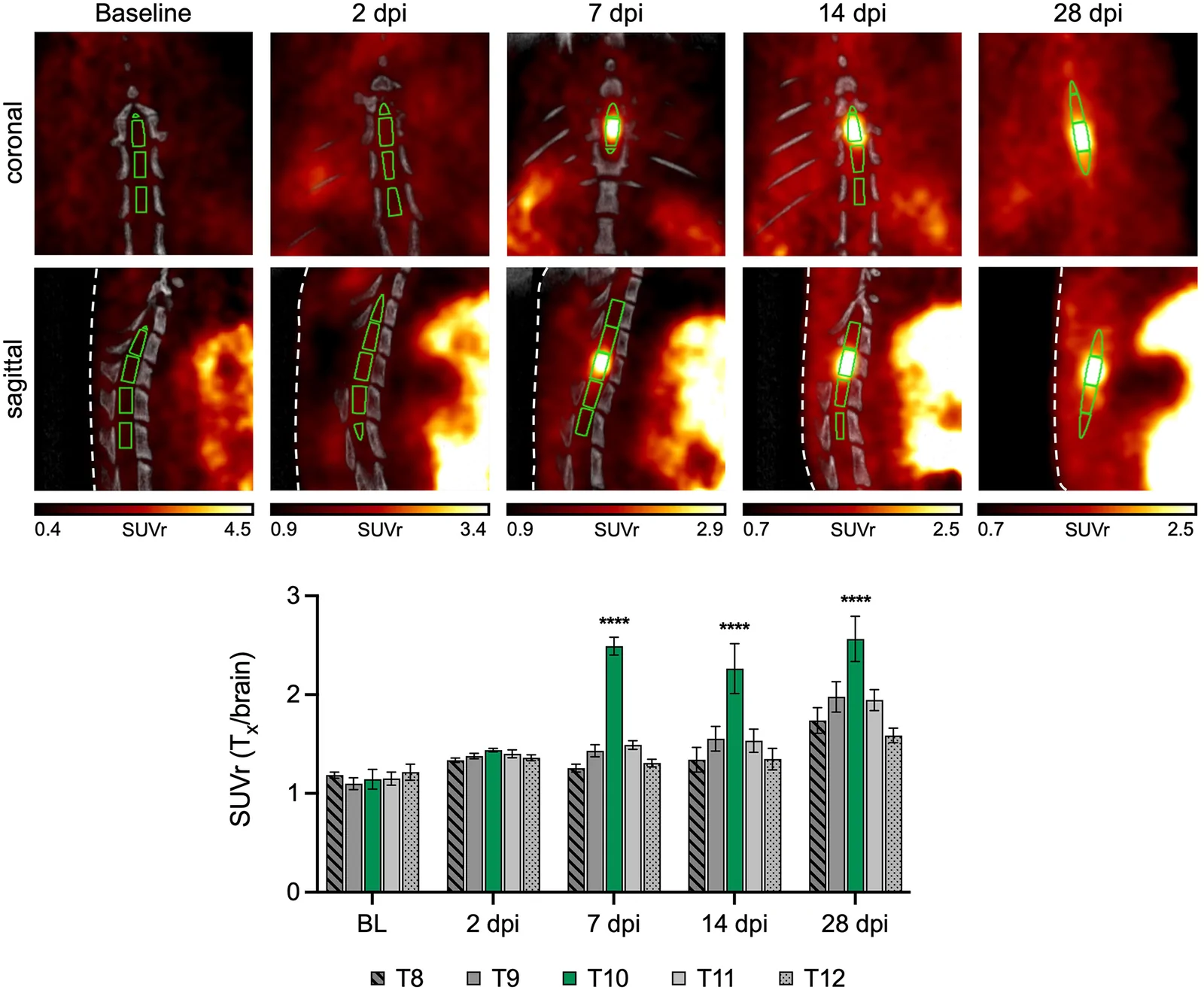
Longitudinal evaluation of [^18^F]3F4AP in rodent SCI. Representative coronal and sagittal views of thoracic [^18^F]3F4AP PET/CT at baseline (BL), 2, 7, 14, and 28 dpi showing CT-based region-of-interest selection for vertebral segments T8–T12. CT was not available for 28 dpi. In this case, region of interest selection was guided by thresholding PET signal. SUVr was quantified by normalization of 10–30-min SUV from injury region of interest (T10, green) and adjacent vertebral segments (T8–T12) to 30–35-min whole-brain SUV. Statistical analysis was performed using a 2-way ANOVA with Dunnett’s multiple comparison test. *Denotes comparison versus BL at T10 (****P < 0.0001). For in vivo PET quantification, *n* = 4 for BL, *n* = 7 for 2 dpi, *n* = 7 for 7 dpi, *n* = 5 for 14 dpi, and *n* = 5 for 28 dpi. Data are mean ± SEM.

After observing high [^18^F]3F4AP uptake at the injury at 7 dpi, we performed [^18^F]3F4AP imaging at multiple time points (baseline, 2, 7, 14, and 28 dpi) to monitor injury progression (Fig. 3). Increased uptake at the injury site (T10) when compared with uptake in the surrounding cord segments was observed at 7, 14, and 28 dpi but not at 2 dpi (SUVr = 1.14 ± 0.10 [baseline, *n* = 4], 1.44 ± 0.02 [2 dpi, *n* = 7], 2.49 ± 0.09 [7 dpi, *n* = 7], 2.26 ± 0.25 [14 dpi, *n* = 5], 2.57 ± 0.23 [28 dpi, *n* = 5]). This suggests the presence of demyelinated fibers after the acute injury phase has subsided. Interestingly, no significant differences in SUVr were observed at the lesion epicenter after the peak at 7 dpi. The T10 SUVr stabilized after 7 dpi, whereas contiguous segments (T9, T11) showed progressive increases at 14 and 28 dpi (Supplemental Fig. 2). Higher SUVr in adjacent spinal levels at later time points indicates that tracer binding continues to increase within a larger volume of the cord as disease progresses, suggesting the spreading of demyelination both caudally and rostrally from the injury. Notably, the stable imaging from day 14 onward is consistent with the plateau observed in the clinical score observed in these animals.

### Ex Vivo Tissue Evaluation Correlates with Imaging and Clinical Assessment

**FIGURE 4.**
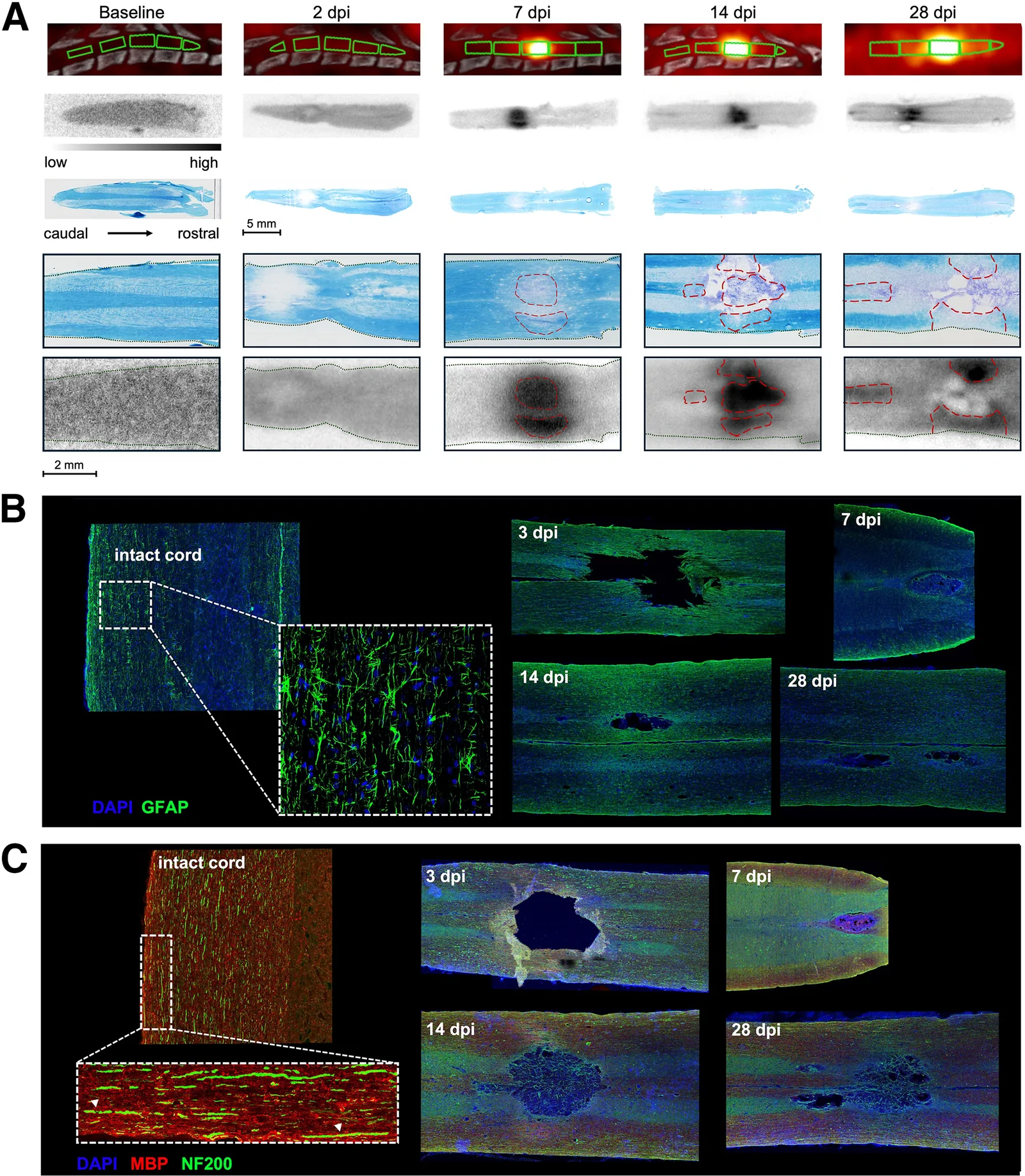
Evaluation of ex vivo SCI tissue. (A) Corresponding PET imaging, autoradiography, and LFB myelin staining of rat spinal cord segments for each time point. Higher-magnification autoradiography and LFB images show tracer uptake and myelin staining in white matter tracts and injury spreading. (B) Representative immunofluorescence staining of intact and injured spinal cord at 3, 7, 14, and 28 dpi with 4′,6-diamidino-2-phenylindole (DAPI; nuclear, blue) and glial fibrillary acidic protein (GFAP; reactive astrocytes, green). (C) Representative immunofluorescence staining of intact and injured spinal cord at 3, 7, 14, and 28 dpi with DAPI (nuclear, blue), myelin basic protein (MBP; myelin basic protein, red), and neurofilament 200 (NF200; axonal marker, green). For immunofluorescence staining, *n* = 1 per time point.

To corroborate that the increased PET signal after injury reflects binding to demyelinated axons rather than other cells or processes, spinal cord tissue was evaluated by ex vivo autoradiography and Luxol Fast Blue (LFB; Alfa Chemistry) myelin staining (Fig. 4). The observed ex vivo autoradiographic signal showed increase in uptake at the lesion site at 7 dpi and after but not at 2 dpi. Since disruption of the blood–spinal cord barrier and infiltration of inflammatory cells are expected to peak 1–2 dpi (55–57), the lack of tracer accumulation at 2 dpi strongly suggests that those processes have minimal effect on the [^18^F]3F4AP signal. Examination of corresponding autoradiography and LFB images supported that the increase in tracer uptake originates from demyelinated white matter tracts around the lesion and not from the gray matter or the cavity. This concurs with binding to demyelinated axons and is inconsistent with binding to infiltrating inflammatory cells or accumulation at the cavity due to changes in blood flow (Fig. 4A). Additionally, although a quantitative autoradiography study on serial tissue sections was not performed, it appears that at later time points (14 and 28 dpi) the area of demyelinated white matter (assessed by fainter LFB staining) extends further from the cavity, indicative of the less focalized injury observed in the PET imaging. Spinal cord tissue from a separate cohort of animals (*n* = 1 per time point) was further assessed for reactive astrocyte, axonal, and myelin content with immunofluorescence staining of glial fibrillary acidic protein, neurofilament 200, and myelin basic protein, respectively (Figs. 4B and 4C). Comparison of intact and injured cord showed a higher density of reactive astrocytes near the injury, as well as greater occurrence of demyelinated fibers near the lesion.

**FIGURE 5.**
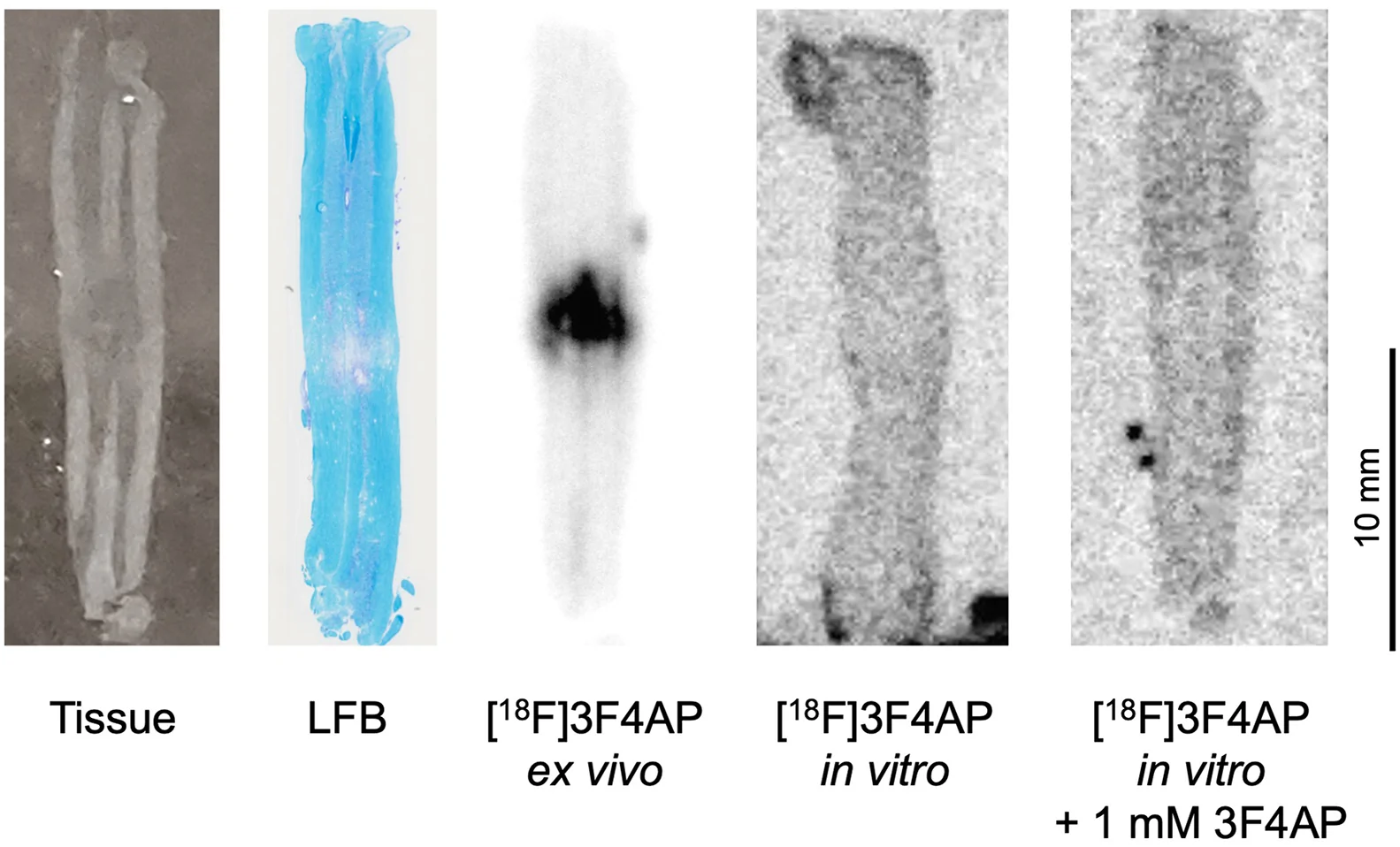
[^18^F]3F4AP specifically binds to exposed K^+^ channels in vivo. Representative tissue photograph, LFB staining, ex vivo autoradiography, in vitro autoradiography, and in vitro autoradiography with blocking dose of 3F4AP. Specific binding in lesion is only observed when tracer is administered to live animal followed by tissue dissection and slicing (ex vivo autoradiography). Incubation of tissue sections with [^18^F]3F4AP in absence or presence of nonradioactive 3F4AP results in no binding, indicating that binding at the lesion in vivo is specific.

To further confirm that the binding of the tracer is specific to K^+^ channels in the injury, in vitro autoradiography was performed on the same fresh-frozen unfixed tissue sections used for ex vivo autoradiography. As shown in Figure 5, tracer binding at the injury was observed only when administered to live animals (ex vivo autoradiography) but not when applied directly to tissue sections (in vitro autoradiography). Tracer application to tissue sections resulted in minimal to no binding at the injury or throughout the tissue. Moreover, addition of an excess amount of nonradioactive 3F4AP did not further decrease binding. This experiment represents a strong indication of specific binding, as it is known that binding to K^+^ channels requires the channels to be in an open conformation (58), which only occurs in living tissue when the membrane is depolarized. If the [^18^F]3F4AP signal were nonspecific, it would be expected to remain in postmortem tissue, which was not observed in this case.

### [^18^F]3F4AP Imaging in Two Human Subjects After SCI

Motivated by the high sensitivity of [^18^F]3F4AP PET imaging in SCI rats, we conducted a pilot study in 2 human subjects with SCIs of different severity and etiologies (Table 1). Both subjects underwent [^18^F]3F4AP PET scans of the injury area, preceded by low-dose CT scans for anatomic reference and attenuation correction. Dynamic PET scans were performed from 0–45 min and 75–106 min to capture images of the initial tracer delivery, providing insights into blood perfusion to the injured cord, as well as images from tracer binding in the injured cord.

**TABLE 1.** Characteristics of Human SCI Volunteers

Subject	Sex	Location	Severity	Interval between injury and scan	Etiology
SCI01	Male	T12	AIS-C (incomplete)	2 y, 5 mo	Fall with traumatic burst fracture
SCI02	Male	T11	AIS-D (incomplete)	7 y, 5 mo	Ruptured spinal cavernous hemangioma

AIS = Asia Impairment Scale.

At the time of the scan, subject SCI01 had an incomplete SCI at T12 (Asia Impairment Scale score = C) as a result from a traumatic fall 2.5 y before imaging and used a wheelchair. The fracture at T12 and the stabilization hardware was visible on the CT (Fig. 6A). Early PET images (0–10 min) revealed the compressed vertebral body and a −76% decrease in PET signal below the injury (L2–L4) compared with above the injury (T10–T11) (Fig. 6B). This reduction in signal is likely due to reduced blood perfusion as indicated by the lower initial peak on the time–activity curves of different spinal segments (Supplemental Fig. 3). Late PET images (75–106 min) showed a moderate reduction in PET signal (−21%) below the injury, which may reflect reduced tracer binding due to partial axonal loss (Fig. 6C).

**FIGURE 6.**
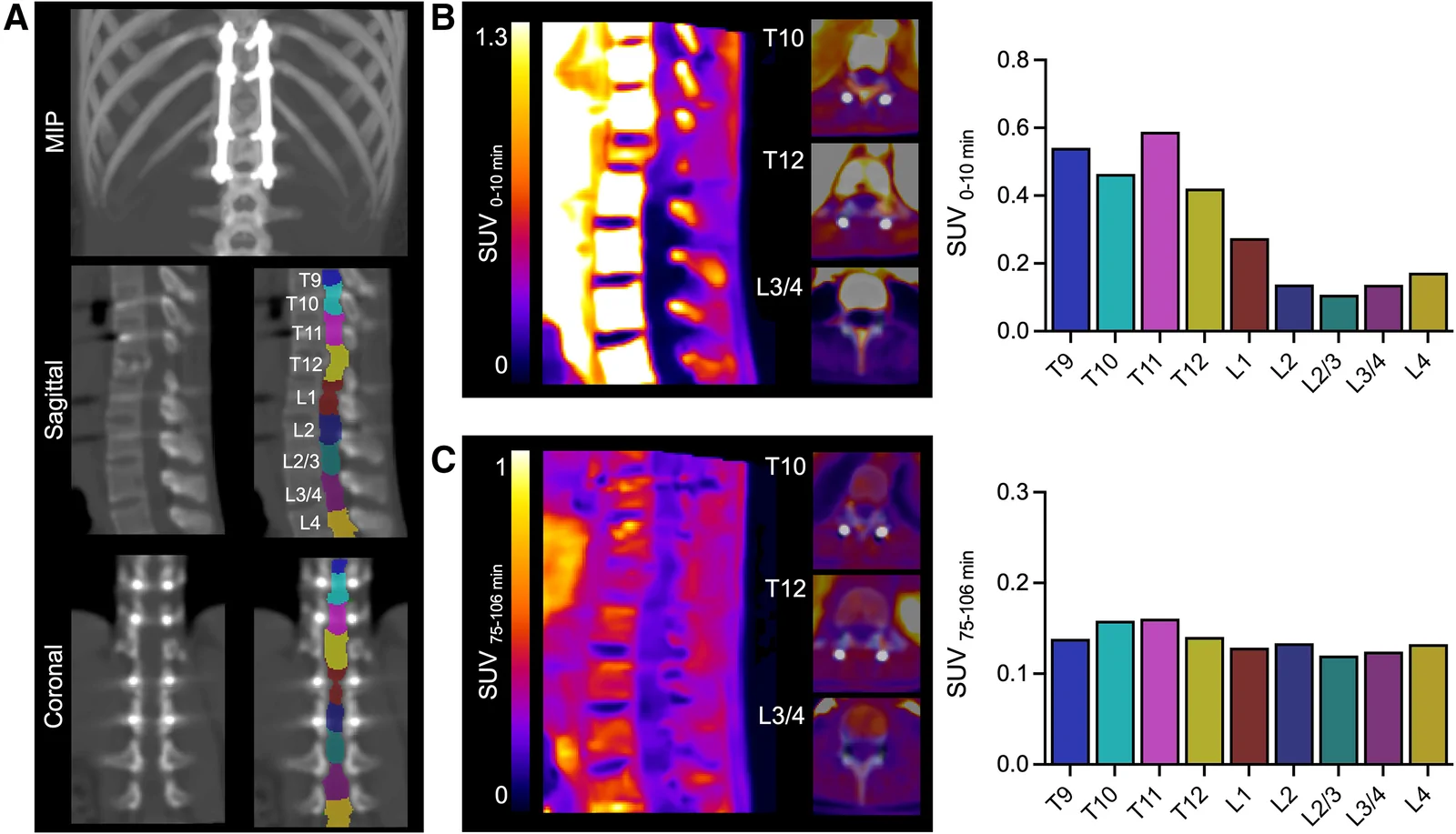
[^18^F]3F4AP in a human subject after severe incomplete SCI. (A) Maximum-intensity projection (MIP), sagittal, and coronal CT images showing location of metal stabilization hardware and crushed vertebral body at T12. Selected regions of interest for segmentation of spinal cord are shown for both sagittal and coronal views. (B) Early PET sagittal images showing compressed vertebral body and low PET signal in cord below injury and quantification 0–10-min SUV at selected spinal segments. (C) Late PET sagittal images showing reduced PET signal in cord below injury and quantification of 75–106-min SUV at selected spinal segments. For both early and late PET, axial images at T10, T12, and L3/4 are shown.

**FIGURE 7.**
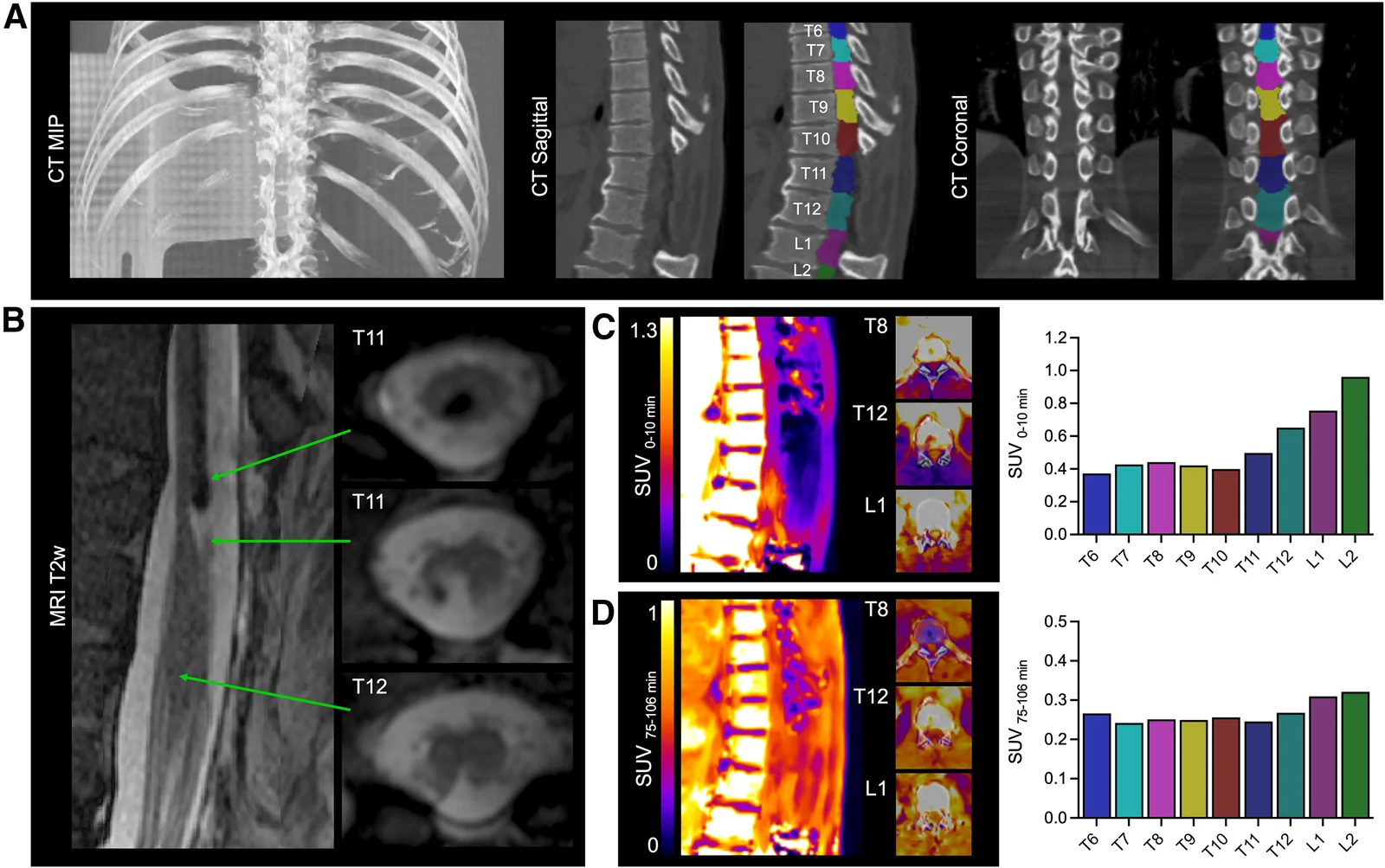
[^18^F]3F4AP in a human subject after mild incomplete SCI. (A) Maximum-intensity projection (MIP), sagittal, and coronal CT images showing laminectomies at T10–T12. Selected regions of interest for segmentation of spinal cord are shown for both sagittal and coronal views. (B) T2-weighted (T2w) MR sagittal and axial images showing spinal lesion. (C) Early PET sagittal images showing high PET signal in cord around injury and quantification 0–10-min SUV at selected spinal segments. (D) Late PET sagittal images showing increased PET signal in cord around injury and quantification of 75–106-min SUV at selected spinal segments. For both early and late PET, axial images at T8, T12, and L1 are shown.

Subject SCI02 had an incomplete SCI at T11 (Asia Impairment Scale score = D) due to a burst spinal hemangioma 7.5 y before imaging, which caused initial paralysis below the injury. After T10–T12 laminectomies (visible on CT; Fig. 7A) and removal of intramedullary lesion, the subject gradually recovered and could walk with minor gait disturbances by the time of the scan. A separate spinal 3 T MRI showed a T2 hyperintense region near the injury compatible with demyelination (Fig. 7B). Early PET images showed a +104% increase in tracer signal below the injury, suggestive of increased blood perfusion likely due to ongoing inflammation (Fig. 7C; Supplemental Fig. 4). Late PET images showed a +26% increase in signal that colocalized with the T2 hyperintensity, consistent with demyelination (Fig. 7D).

## DISCUSSION

This study demonstrates that [^18^F]3F4AP is highly sensitive to incomplete SCI in rats. PET imaging in a rat spinal contusion model revealed more than a 2-fold increase in tracer binding, highly localized to the injured spinal segment compared with baseline. This result aligns with previous studies in similar models, which demonstrate axonal sparing with severe demyelination and upregulation of axonal K^+^ channels at the injury site starting around 7 dpi (44,45). Notably, the [^18^F]3F4AP signal evolved over time. In the acute SCI phase (2 dpi), no significant increase in tracer uptake was observed, suggesting that damaged myelin has not yet been cleared and axonal K_v_ channels have not redistributed. This is consistent with Karimi-Abdoolrezaee’s findings in a compressive SCI model, where this process occurs at later, more chronic stages of SCI rather than as an early response to injury. This finding also supports that the [^18^F]3F4AP signal is not dependent on blood–spinal cord barrier damage or inflammation, which are expected to peak very soon after injury. By 1 wk after injury, the signal reached its maximal intensity, aligning with the peak of demyelination. Subsequently, at 14 and 28 dpi, the signal began to spread out, consistent with the progression of demyelination.

Agreement between PET, autoradiography, and LFB myelin staining supports the notion that the [^18^F]3F4AP signal primarily originates from demyelinated axons. Ex vivo autoradiography and LFB staining of paired tissue sections show that [^18^F]3F4AP primarily localizes to demyelinated white matter bands, not to the cavity or gray matter. Representative immunohistochemistry also corroborated a high concentration of demyelinated axons at 7, 14, and 28 dpi. Furthermore, in vitro autoradiography on the same tissue sections showed no binding in the injured tissue, reinforcing that the binding is specific to K^+^ channels.

Many human SCIs are incomplete, with some spared circuits that hold potential for recovery of voluntary movement (59). Slower and weaker axonal conduction after demyelination has been suggested as a pathophysiologic basis for discomplete SCIs, characterized by apparent complete transection as judged by clinical criteria but with neurophysiologic evidence of conduction through the level of damage (2,60,61). Our pilot imaging study in 2 human SCI subjects represents a crucial step in the translational potential of [^18^F]3F4AP. The study participant with a burst spinal hemanginoma (SCI02) experienced near-complete recovery, whereas the study participant with a traumatic fall injury (SCI01) had initial gains in the first month after injury from Asia Impairment Scale A to Asia Impairment Scale C, but muscle strength and sensation below the injury remained low. Thus, these 2 participants represent a broad range in terms of the potential to have residual axons crossing the spinal lesion. The milder case showed increased binding at and immediately below the injury, suggestive of the presence of demyelinated fibers. The smaller increase in tracer binding in this subject compared with the rat SCI injury model may reflect limited demyelination in this particular subject given the nature of the injury and the extended period (7.5 y) between injury and scan. Conversely, the more severe case exhibited a decrease in binding at and below the injury. These initial observations are promising, but larger cohort studies are warranted to validate and generalize these trends.

This study has limitations. First, PET acquisition protocols differed between humans and rats (i.e., injection on the scanner vs. injection on the bench), allowing tracer delivery monitoring in humans but not in rats. Second, to compare across animals and time points, we normalized by brain SUV, which may overlook brain-specific or global changes in tracer binding or distribution outside the injured cord that may arise because of the injury. Future studies should evaluate changes distal to the injury. Additionally, injury-to-imaging timeline varied: 2 to 28 d in rats and 2.5 and 7.5 y in humans, capturing the acute, subacute, and chronic injury phases in animals but only the chronic stage in humans. Although SCI is more prevalent in men, we used only female rats because of easier management of postinjury bladder dysfunction. Lastly, whereas previous studies have documented K_v_ channel changes in similar models, in this study, we did not directly measure these changes in animal or human tissue samples by immunohistochemistry.

Given the heterogeneity of SCI, a tracer capable of detecting spared axons is of paramount importance. Our study highlights [^18^F]3F4AP’s value in detecting demyelination and axonal damage, providing valuable insights into SCI pathology. Although some clinical studies have shown the benefits of using 4AP in SCI patients (35–40), others have not (41–43), raising questions about the role of demyelination in certain cases. This emphasizes the need for a biomarker that can predict response to 4AP. Our findings lay the groundwork for validating [^18^F]3F4AP as a quantitative biomarker for diagnosing, prognosticating, and evaluating SCI treatment efficacy, in contrast to the current neurologic assessments used in clinical trials, which may be inaccurate as they reflect only the present state and not the potential for recovery. [^18^F]3F4AP could serve as a sensitive and quantitative biomarker in clinical trials by measuring spared demyelinated axons with recovery potential.

## CONCLUSION

Our study represents the first investigation of [^18^F]3F4AP in traumatic SCI, demonstrating its high sensitivity and specificity for detecting demyelinated axons. Further studies with larger cohorts and longitudinal assessments are crucial to confirm the reliability of [^18^F]3F4AP PET as a diagnostic tool. Additionally, exploring its role in monitoring therapeutic interventions and its broader applicability in diverse SCI populations will be essential for clinical translation. These promising results lay the foundation for future investigations into the clinical utility of [^18^F]3F4AP in SCI.

## DISCLOSURE

Pedro Brugarolas is a named inventor on patents related to [^18^F]3F4AP owned by the University of Chicago. Pedro Brugarolas’ interests were reviewed and are managed by Massachusetts General Hospital and Massachusetts General Brigham in accordance with their conflict-of-interest policies. Additional funding was received from a Translational Research Award from Boston Children’s Hospital (to Zhigang He and Pedro Brugarolas); the Ellen R. and Melvin J. Gordon Center for the Cure and Treatment of Paralysis Pilot Grant at Spaulding Rehabilitation Hospital (to Clas Linnman); National Institutes of Health grant R01NS114066 (to Pedro Brugarolas); the Massachusetts General Hospital Executive Committee on Research Physician Scientist Development Award (to Karla Ramos-Torres); and National Institutes of Health grant K99EB033407 (to Yu-Peng Zhou). The datasets generated or analyzed during the current study are available from the corresponding author on reasonable request. A preprint version of this article was posted on bioRxiv on April 28, 2024: https://doi.org/10.1101/2024.04.24.590984. No other potential conflict of interest relevant to this article was reported.

## Notes

KEY POINTS **QUESTION:** Can [^18^F]3F4AP PET be used to image demyelinated axons present after SCI? **PERTINENT FINDINGS:** Rats with spinal contusion injuries were imaged with [^18^F]3F4AP up to 1 mo after injury. A 2.1-fold higher [^18^F]3F4AP uptake was observed in the injured cord segment at 7 dpi compared with baseline. Good agreement between PET findings and autoradiography, LFB staining for myelin, and immunohistochemistry markers support the conclusion that the [^18^F]3F4AP signal primarily originates from demyelinated axons. In a pilot study with 2 human subjects [^18^F]3F4AP PET differentiated between incomplete injuries of different severities, indicating the presence of different physiologic processes such as axonal loss and demyelination in each. **IMPLICATIONS FOR PATIENT CARE:** The promising findings presented here lay the foundation for future investigations into the clinical utility of [^18^F]3F4AP as a diagnostic or therapeutic monitoring tool in the field of SCI.

## References

[1] Ziegler G Grabher P Thompson A. Progressive neurodegeneration following spinal cord injury: implications for clinical trials. Neurology. 2018;90:e1257–e1266. 29514946 10.1212/WNL.0000000000005258 PMC5890610

[2] Waxman SG. Demyelination in spinal cord injury. J Neurol Sci. 1989;91:1–14. 2664092 10.1016/0022-510x(89)90072-5

[3] Freund P Weiskopf N Ashburner J. MRI investigation of the sensorimotor cortex and the corticospinal tract after acute spinal cord injury: a prospective longitudinal study. Lancet Neurol. 2013;12:873–881. 23827394 10.1016/S1474-4422(13)70146-7 PMC3744750

[4] McDonald JW Belegu V. Demyelination and remyelination after spinal cord injury. J Neurotrauma. 2006;23:345–359. 16629621 10.1089/neu.2006.23.345

[5] Franklin RJ Ffrench-Constant C. Remyelination in the CNS: from biology to therapy. Nat Rev Neurosci. 2008;9:839–855. 18931697 10.1038/nrn2480

[6] Franklin RJ Kotter MR. The biology of CNS remyelination: the key to therapeutic advances. J Neurol. 2008;255(suppl 1):19–25. 10.1007/s00415-008-1004-6 18317673

[7] Mi S Miller RH Tang W. Promotion of central nervous system remyelination by induced differentiation of oligodendrocyte precursor cells. Ann Neurol. 2009;65:304–315. 19334062 10.1002/ana.21581

[8] Kotter MR Stadelmann C Hartung HP. Enhancing remyelination in disease—can we wrap it up? Brain. 2011;134:1882–1900. 21507994 10.1093/brain/awr014

[9] Münzel EJ Williams A. Promoting remyelination in multiple sclerosis-recent advances. Drugs. 2013;73:2017–2029. 24242317 10.1007/s40265-013-0146-8 PMC3853368

[10] Keough MB Yong VW. Remyelination therapy for multiple sclerosis. Neurotherapeutics. 2013;10:44–54. 23070731 10.1007/s13311-012-0152-7 PMC3557365

[11] Franklin RJ Gallo V. The translational biology of remyelination: past, present, and future. Glia. 2014;62:1905–1915. 24446279 10.1002/glia.22622

[12] Plemel JR Keough MB Duncan GJ. Remyelination after spinal cord injury: is it a target for repair? Prog Neurobiol. 2014;117:54–72. 24582777 10.1016/j.pneurobio.2014.02.006

[13] Brugarolas P Popko B. Remyelination therapy goes to trial for multiple sclerosis. Neurol Neuroimmunol Neuroinflamm. 2014;1:e26. 25340076 10.1212/NXI.0000000000000026 PMC4202671

[14] Najm FJ Madhavan M Zaremba A. Drug-based modulation of endogenous stem cells promotes functional remyelination in vivo. Nature. 2015;522:216–220. 25896324 10.1038/nature14335 PMC4528969

[15] Kremer D Kury P Dutta R. Promoting remyelination in multiple sclerosis: current drugs and future prospects. Mult Scler. 2015;21:541–549. 25623245 10.1177/1352458514566419

[16] Ahuja CS Wilson JR Nori S. Traumatic spinal cord injury. Nat Rev Dis Primers. 2017;3:17018. 28447605 10.1038/nrdp.2017.18

[17] Mallik S Samson RS Wheeler-Kingshott CA Miller DH. Imaging outcomes for trials of remyelination in multiple sclerosis. J Neurol Neurosurg Psychiatry. 2014;85:1396–1404. 24769473 10.1136/jnnp-2014-307650 PMC4335693

[18] Jungmann PM Agten CA Pfirrmann CW Sutter R. Advances in MRI around metal. J Magn Reson Imaging. 2017;46:972–991. 28342291 10.1002/jmri.25708

[19] Stroman PW Wheeler-Kingshott C Bacon M. The current state-of-the-art of spinal cord imaging: methods. Neuroimage. 2014;84:1070–1081. 23685159 10.1016/j.neuroimage.2013.04.124 PMC4371133

[20] von Leden RE Selwyn RG Jaiswal S Wilson CM Khayrullina G Byrnes KR. ^18^F-FDG-PET imaging of rat spinal cord demonstrates altered glucose uptake acutely after contusion injury. Neurosci Lett. 2016;621:126–132. 27084688 10.1016/j.neulet.2016.04.027 PMC5018212

[21] Tremoleda JL Thau-Zuchman O Davies M. In vivo PET imaging of the neuroinflammatory response in rat spinal cord injury using the TSPO tracer [^18^F]GE-180 and effect of docosahexaenoic acid. Eur J Nucl Med Mol Imaging. 2016;43:1710–1722. 27154521 10.1007/s00259-016-3391-8 PMC4932147

[22] Fang H Rossano S Wang X. Translational PET imaging of spinal cord injury with the serotonin transporter tracer [^11^C]AFM. Mol Imaging Biol. 2022;24:560–569. 35020138 10.1007/s11307-021-01698-7 PMC9550197

[23] Bertoglio D Halloin N Lombaerde S. SV2A PET imaging is a noninvasive marker for the detection of spinal damage in experimental models of spinal cord injury. J Nucl Med. 2022;63:1245–1251. 35027368 10.2967/jnumed.121.263222 PMC9364338

[24] Wu C Zhu J Baeslack J. Longitudinal positron emission tomography imaging for monitoring myelin repair in the spinal cord. Ann Neurol. 2013;74:688–698. 23818306 10.1002/ana.23965

[25] Wu C Eck B Zhang S. Discovery of 1,2,3-triazole derivatives for multimodality PET/CT/cryoimaging of myelination in the central nervous system. J Med Chem. 2017;60:987–999. 28107629 10.1021/acs.jmedchem.6b01328

[26] Tiwari AD Zhu J You J. Novel ^18^F-labeled radioligands for positron emission tomography imaging of myelination in the central nervous system. J Med Chem. 2019;62:4902–4914. 31042384 10.1021/acs.jmedchem.8b01354

[27] Brugarolas P Sanchez-Rodriguez JE Tsai HM. Development of a PET radioligand for potassium channels to image CNS demyelination. Sci Rep. 2018;8:607. 29330383 10.1038/s41598-017-18747-3 PMC5766510

[28] Guehl NJ Ramos-Torres KM Linnman C. Evaluation of the potassium channel tracer [^18^F]3F4AP in rhesus macaques. J Cereb Blood Flow Metab. 2021;41:1721–1733. 33090071 10.1177/0271678X20963404 PMC8221756

[29] Brugarolas P Wilks MQ Noel J. Human biodistribution and radiation dosimetry of the demyelination tracer [^18^F]3F4AP. Eur J Nucl Med Mol Imaging. 2023;50:344–351. 36197499 10.1007/s00259-022-05980-w PMC9816249

[30] Lewis MJ Laber E Olby NJ. Predictors of response to 4-aminopyridine in chronic canine spinal cord injury. J Neurotrauma. 2019;36:1428–1434. 30235970 10.1089/neu.2018.5975 PMC6482892

[31] Gruner JA Yee AK. 4-Aminopyridine enhances motor evoked potentials after graded spinal cord compression injury in rats. Brain Res. 1999;816:446–456. 9878868 10.1016/s0006-8993(98)01184-6

[32] Blight AR. Effect of 4-aminopyridine on axonal conduction-block in chronic spinal cord injury. Brain Res Bull. 1989;22:47–52. 2540887 10.1016/0361-9230(89)90126-3

[33] Blight AR Gruner JA. Augmentation by 4-aminopyridine of vestibulospinal free fall responses in chronic spinal-injured cats. J Neurol Sci. 1987;82:145–159. 2831307 10.1016/0022-510x(87)90014-1

[34] Blight AR Toombs JP Bauer MS Widmer WR. The effects of 4-aminopyridine on neurological deficits in chronic cases of traumatic spinal cord injury in dogs: a phase I clinical trial. J Neurotrauma. 1991;8:103–119. 1870134 10.1089/neu.1991.8.103

[35] Hayes KC Blight AR Potter PJ. Preclinical trial of 4-aminopyridine in patients with chronic spinal cord injury. Paraplegia. 1993;31:216–224. 8493036 10.1038/sc.1993.40

[36] Hansebout RR Blight AR Fawcett S Reddy K. 4-Aminopyridine in chronic spinal cord injury: a controlled, double-blind, crossover study in eight patients. J Neurotrauma. 1993;10:1–18. 8320728 10.1089/neu.1993.10.1

[37] Segal JL Pathak MS Hernandez JP Himber PL Brunnemann SR Charter RS. Safety and efficacy of 4-aminopyridine in humans with spinal cord injury: a long-term, controlled trial. Pharmacotherapy. 1999;19:713–723. 10391417 10.1592/phco.19.9.713.31540

[38] Wolfe DL Hayes KC Hsieh JT Potter PJ. Effects of 4-aminopyridine on motor evoked potentials in patients with spinal cord injury: a double-blinded, placebo-controlled crossover trial. J Neurotrauma. 2001;18:757–771. 11526982 10.1089/089771501316919120

[39] Grijalva I Guizar-Sahagun G Castaneda-Hernandez G. Efficacy and safety of 4-aminopyridine in patients with long-term spinal cord injury: a randomized, double-blind, placebo-controlled trial. Pharmacotherapy. 2003;23:823–834. 12885095 10.1592/phco.23.7.823.32731

[40] Grijalva I Garcia-Perez A Diaz J. High doses of 4-aminopyridine improve functionality in chronic complete spinal cord injury patients with MRI evidence of cord continuity. Arch Med Res. 2010;41:567–575. 21167397 10.1016/j.arcmed.2010.10.001

[41] van der Bruggen MA Huisman HB Beckerman H Bertelsmann FW Polman CH Lankhorst GJ. Randomized trial of 4-aminopyridine in patients with chronic incomplete spinal cord injury. J Neurol. 2001;248:665–671. 11569894 10.1007/s004150170111

[42] DeForge D Nymark J Lemaire E. Effect of 4-aminopyridine on gait in ambulatory spinal cord injuries: a double-blind, placebo-controlled, crossover trial. Spinal Cord. 2004;42:674–685. 15356676 10.1038/sj.sc.3101653

[43] Cardenas DD Ditunno JF Graziani V. Two phase 3, multicenter, randomized, placebo-controlled clinical trials of fampridine-SR for treatment of spasticity in chronic spinal cord injury. Spinal Cord. 2014;52:70–76. 24216616 10.1038/sc.2013.137

[44] Nashmi R Fehlings MG. Mechanisms of axonal dysfunction after spinal cord injury: with an emphasis on the role of voltage-gated potassium channels. Brain Res Brain Res Rev. 2001;38:165–191. 11750932 10.1016/s0165-0173(01)00134-5

[45] Karimi-Abdolrezaee S Eftekharpour E Fehlings MG. Temporal and spatial patterns of Kv1.1 and Kv1.2 protein and gene expression in spinal cord white matter after acute and chronic spinal cord injury in rats: implications for axonal pathophysiology after neurotrauma. Eur J Neurosci. 2004;19:577–589. 14984408 10.1111/j.0953-816x.2004.03164.x

[46] Sotome A Kadoya K Suzuki Y Iwasaki N. Spinal canal and spinal cord in rat continue to grow even after sexual maturation: anatomical study and molecular proposition. Int J Mol Sci. 2022;23:16076. 36555713 10.3390/ijms232416076 PMC9781254

[47] Wang G Qi J. PET image reconstruction using kernel method. IEEE Trans Med Imaging. 2015;34:61–71. 25095249 10.1109/TMI.2014.2343916 PMC4280333

[48] Spangler-Bickell MG Deller TW Bettinardi V Jansen F. Ultra-fast list-mode reconstruction of short PET frames and example applications. J Nucl Med. 2021;62:287–292. 32646873 10.2967/jnumed.120.245597

[49] James ND Bartus K Grist J Bennett DL McMahon SB Bradbury EJ. Conduction failure following spinal cord injury: functional and anatomical changes from acute to chronic stages. J Neurosci. 2011;31:18543–18555. 22171053 10.1523/JNEUROSCI.4306-11.2011 PMC3495307

[50] Basso DM Beattie MS Bresnahan JC. A sensitive and reliable locomotor rating scale for open field testing in rats. J Neurotrauma. 1995;12:1–21. 7783230 10.1089/neu.1995.12.1

[51] Basuli F Zhang X Brugarolas P Reich DS Swenson RE. An efficient new method for the synthesis of 3-[^18^F]fluoro-4-aminopyridine via Yamada-Curtius rearrangement. J Labelled Comp Radiopharm. 2018;61:112–117. 28870001 10.1002/jlcr.3560 PMC5992582

[52] Ramos-Torres K Noel J Vesper D Rice P Brugarolas P Yokell D. cGMP production of [^18^F]3F4AP for human PET imaging. J Nucl Med. 2021;62(suppl 1):1461.33741642

[53] Kjell J Olson L. Rat models of spinal cord injury: from pathology to potential therapies. Dis Model Mech. 2016;9:1125–1137. 27736748 10.1242/dmm.025833 PMC5087825

[54] Ramos-Torres K Sun Y Takahashi K Zhou YP Brugarolas P. Common anesthetic used in preclinical PET imaging inhibits metabolism of the PET tracer [^18^F]3F4AP. J Neurochem. 2024;168:2577–2586. 38690718 10.1111/jnc.16118 PMC11482445

[55] Hellenbrand DJ Quinn CM Piper ZJ Morehouse CN Fixel JA Hanna AS. Inflammation after spinal cord injury: a review of the critical timeline of signaling cues and cellular infiltration. J Neuroinflammation. 2021;18:284. 34876174 10.1186/s12974-021-02337-2 PMC8653609

[56] Freyermuth-Trujillo X Segura-Uribe JJ Salgado-Ceballos H Orozco-Barrios CE Coyoy-Salgado A. Inflammation: a target for treatment in spinal cord injury. Cells. 2022;11:2692. 36078099 10.3390/cells11172692 PMC9454769

[57] Kwiecien JM Dabrowski W Dąbrowska-Bouta B. Prolonged inflammation leads to ongoing damage after spinal cord injury. PLoS One. 2020;15:e0226584. 32191733 10.1371/journal.pone.0226584 PMC7081990

[58] Choquet D Korn H. Mechanism of 4-aminopyridine action on voltage-gated potassium channels in lymphocytes. J Gen Physiol. 1992;99:217–240. 1613484 10.1085/jgp.99.2.217 PMC2216608

[59] Raineteau O Schwab ME. Plasticity of motor systems after incomplete spinal cord injury. Nat Rev Neurosci. 2001;2:263–273. 11283749 10.1038/35067570

[60] Awad A Levi R Waller M Westling G Lindgren L Eriksson J. Preserved somatosensory conduction in complete spinal cord injury: discomplete SCI. Clin Neurophysiol. 2020;131:1059–1067. 32197128 10.1016/j.clinph.2020.01.017

[61] Wrigley PJ Siddall PJ Gustin SM. New evidence for preserved somatosensory pathways in complete spinal cord injury: a fMRI study. Hum Brain Mapp. 2018;39:588–598. 29080262 10.1002/hbm.23868 PMC6866574

